# Cases of Induration in the Corpus-Cavernosum

**Published:** 1867-07

**Authors:** H. V. M. Miller

**Affiliations:** Prof. of Obstetrics in Atlanta Medical College


### ARTICLE IV.

*Cases of Induration in the Corpus-Cavernosum.* By H. V. M. Miller, M. D., Prof. of Obstetrics in Atlanta Medical College.

Subjoined is a brief account of three cases of an interesting lesion of the corpus-cavernosum.

It is probably of rare occurrence, since these are the only cases which the writer has observed in many years of practice. Books, it is believed, contain no information in regard to it; and none of the medical men to whom it has been mentioned remember to have noticed it.

A gentleman greatly distinguished as a jurist and a wit, complained of some alteration of the structure of his penis, which he feared and was advised might be incipient cancer. Upon examination, a small induration could be felt on the dorsum of the penis imbedded in the substance of the corpus-cavernosum. The induration, when compressed with the finger, appeared to be about an inch in length, and of half that breadth; there was no elevation of the surface; the skin moved freely over the indurated part; there was no increase of heat and no tenderness when the organ was flaccid; but the erections were more or less painful, as they were more or less complete, and always accompanied by an upward curvature of the organ. It was bent in the opposite direction to what takes place in chordee, and at a sharper angle, and to such an extent as seriously to interfere with copulation. The gentleman was more than sixty years old, had not had gonorrhœa at any period of his life, and though at that time a widower, had lived forty years in wedlock, and could not remember that he had received any local injury of the part. There was no reason to suppose that the induration was malignant; but in view of the age of the patient, it was thought more probable that the erectile tissue, of which the corpus-cavernosum in part consists, had so lost its contractility, that the accumulated blood on which the erection depends, had not been properly expelled from the cavernous cells in which it had been effused when the orgasm had subsided. This being repeated from time to time, the fibrine of the blood had finally coagulated, become organized, and glued together some of the cells, thus rendering that portion of the organ incapable of distention, and consequently of participation in the erection, so that when the unaffected cells became filled with blood, there was necessarily curvature upwards, as it occurs in the opposite direction in chordee. The difference between the two conditions being, that in the latter the effusion occurs in the corpus spungiosum, and is the result of acute inflammation, while in the former a different structure was affected without preceding inflammatory action, or, if any, of a chronic character. Acting upon this hypothesis, tincture of iodine was penciled upon the penis, alterative doses of mercury given for a few weeks, followed by iodide of potassium for several months, in the hope that the effused fibrine might be dissolved and absorbed, and the discussion of the induration thus secured. Happily, these results followed: the tumor became gradually smaller, and the curvature on erection less noticeable.

The gentleman subsequently married, and nothing more was heard of the case.

The second example occurred in a man also advanced in life, probably sixty years old, many years married, but boasting no perfect fidelity to the nuptial vow. Once or twice in his life he had contracted gonorrhœa, but not within the last ten years.

The induration in his case was situated about midway between the symphysis of the pubis and the glans penis, but not centrally upon the dorsum: it was confined to one lateral half of the penis; the skin moved freely above it; there was slight elevation of the surface; and it felt, when compressed, like a hard oval body about the size of a large filbert, imbedded in the substance of the corpus-cavernosum. When erections came on, the curvature was not directly upwards, but more laterally, and, to a considerable extent, rendering copulation difficult and painful by the divergence of the organ from the right line, and also by the pain which occurred at the seat of the induration. He stated that some months previously, he had received a hurt or contusion on the organ, and that the point where the curvature occurred had been tender ever since, and still does not bear compression without suffering.

This case differed from the first in its seat, in its supposed production by violence, and by the evidence of some existing inflammatory action; there seemed, however, no sufficient reason to conclude that there was any real pathological difference, so the same general treatment was recommended, preceding the use of the tincture of iodine by a blister along the dorsum, and after this had healed, the remedies employed were in both cases alike. The termination in this was not so favorable as in the first case. After three or four months assiduous use of the means recommended, the tenderness had disappeared; there was no increase of effusion, but the hardness still remained, and the lateral upward curvature was as marked and annoying as before. The case at this period of its history was lost sight of.

The third instance occurred in a gentleman fifty-three years old, and never married. He has frequently had gonorrhœa, but not within several years of the time when he first noticed the tumors, as he called them, upon his penis. Upon examination, there was found about an inch from the symphysis, on the dorsum of the penis, a flattened induration in the substance of the corpus-cavernosum, apparently about as large as a butter bean. At the distance of an inch behind the glans penis, another could be felt nearly of the same size, but seemingly confined to the left lateral half of the organ. No tenderness was felt in either of them: they gave no trouble whatever when the penis was flaccid; but as often as erections happened, more or less of tension and discomfort was experienced, but no great suffering. They were not preceded by any injury that he remembered, and their formation had been accompanied by no inflammatory symptoms whatever. The distortion of the penis in erection was very remarkable: the portion of it nearest to the body was bent upwards as in the first case above described; but the anterior third ot it was drawn quite to one side by a lateral curvature, giving to the entire organ quite an unusual and somewhat ridiculous contour.

If the opinions expressed above of the pathological anatomy of this lesion be correct, this peculiar shape of the penis in erection becomes quite intelligible: it could assume no other. Upon the correctness of these opinions, has been based whatever of treatment has been attempted in these cases—apparently successful in the first, and but partially so in the second; though it is to be remembered that the means employed, had not been used sufficiently long to determine that they would not ultimately have succeeded. Fibrinous effusions in other parts of the body where they become organized are of difficult solution, and it often requires many month's use of any of the alteratives at our command, to secure this result. The third case is still under treatment, and its termination of course, conjectural.

It will be remarked, that all these patients were men advanced in life, and in none of them could the metamorphosis of tissue be fairly attributed to existing or preceding venereal disease, and in but one of them was any injury of the organ remembered, to which the growth could by possibility be attributed. When it is recollected how much more liable to injury this organ is in the young than in the old, and how common are attacks of venereal diseases at different periods of life, and yet neither as the sequel of one or the other of them, has this peculiar lesion been remarked in a single instance, we are led reasonably to conclude that it does not depend upon either for its formation. A review of the history of all three of the cases justifies the opinion formed upon the examination of the first of them. A knowledge of the structure of the organ, and its physiological action, raises the presumption, that such deposits are likely to occur only in the aged, and though exceedingly rare in them, may be regarded as peculiar to that period of life.

There is no good reason for mistake in the diagnosis of this injury. Still, in the first of the above cases, it was sus pected to be cancerous; in the second, it was actually treated as a stricture of the urethra, though, there was no diminution of the caliber of that opening, and not the slightest difficulty in voiding the urine; in the third case it had been proposed to remove the induration with the knife, and this would probably have been done if there had happened to be but one of them on the organ. It is not easy to perceive how such an operation would be likely to restore the normal condition of the penis: the induration could doubtless be removed, and the wound would heal, but the possibility of perfect erection would at the same time be destroyed.

As it is a disease not likely to terminate fatally, occurring late in life, and productive only of discomfort and annoyance, painful operative procedures, even if they promised success, ought not to be lightly undertaken.

